# A bivalent *β*-carboline derivative inhibits macropinocytosis-dependent entry of pseudorabies virus by targeting the kinase DYRK1A

**DOI:** 10.1016/j.jbc.2023.104605

**Published:** 2023-03-12

**Authors:** Chongyang Wang, Ruochen Hu, Ting Wang, Liuyuan Duan, Qili Hou, Junru Wang, Zengqi Yang

**Affiliations:** 1 College of Veterinary Medicine, Northwest A&F University, Xianyang, China; 2 College of Chemistry and Pharmacy, Northwest A&F University, Xianyang, China

**Keywords:** antiviral, pseudorabies virus, *β*-carboline, dual-specificity tyrosine phosphorylation–regulated kinase 1A, macropinocytosis

## Abstract

Pseudorabies virus (PRV) has become a “new life-threatening zoonosis” since the human-originated PRV strain was first isolated in 2020. To identify novel anti-PRV agents, we screened a total of 107 *β*-carboline derivatives and found 20 compounds displaying antiviral activity against PRV. Among them, 14 compounds showed better antiviral activity than acyclovir. We found that compound **45** exhibited the strongest anti-PRV activity with an IC_50_ value of less than 40 nM. Our *in vivo* studies showed that treatment with **45** significantly reduced the viral loads and protected mice challenged with PRV. To clarify the mode of action of **45**, we conducted a time of addition assay, an adsorption assay, and an entry assay. Our results indicated that **45** neither had a virucidal effect nor affected viral adsorption while significantly inhibiting PRV entry. Using the FITC–dextran uptake assay, we determined that **45** inhibits macropinocytosis. The actin-dependent plasma membrane protrusion, which is important for macropinocytosis, was also suppressed by **45**. Furthermore, the kinase DYRK1A (dual-specificity tyrosine phosphorylation–regulated kinase 1A) was predicted to be a potential target for **45**. The binding of **45** to DYRK1A was confirmed by drug affinity responsive target stability and cellular thermal shift assay. Further analysis revealed that knockdown of DYRK1A by siRNA suppressed PRV macropinocytosis and the tumor necrosis factor alpha-TNF–induced formation of protrusions. These results suggested that **45** could restrain PRV macropinocytosis by targeting DYRK1A. Together, these findings reveal a unique mechanism through which *β*-carboline derivatives restrain PRV infection, pointing to their potential value in the development of anti-PRV agents.

Pseudorabies (PR; also called Aujeszky’s disease) is caused by pseudorabies virus (PRV; also known as Aujeszky’s disease virus or suid herpesvirus type 1), which belongs to the *Alphaherpesvirinae* subfamily of the family Herpesviridae. PRV is a highly contagious pathogen that primarily infects swine, and PRV infection could induce reproductive failure in sows and fatal encephalitis in newborn piglets. Vaccination with the Bartha-K61 strain was an effective method to control PR in China, until new PRV variant strains emerged in 2011. Since then, the second PR outbreak has caused significant economic losses in the swine industry (1, 2). In addition to the natural host, PRV can infect many mammals including goats, cattle, sheep, and dogs (3). Although cases of humans infected with PRV had been occasionally reported, human susceptibility remained controversial (4). However, since 2017, multiple cases of PRV causing endophthalmitis and encephalitis in humans have been reported, indicating that PRV could infect humans (5, 6). In 2020, a human-originated PRV strain was first isolated from an acute human encephalitis case. This strain had genetic characteristics that were similar to those of current Chinese variant strains (7). These reports highlight the great risk of PRV transmission from pigs to humans. Thus, there is an urgent need to discover antiviral drugs against PRV.

Screening natural products is a well-established method to obtain potential drugs with therapeutic activity. *β*-Carboline alkaloids are distributed in a wide range of sources, including plants, mammals, and microorganisms. Moreover, *β*-carbolines display a large spectrum of biological activities, such as antitumor, antiparasitic, anxiolytic, and antiviral effects (8). Harmine, a well-studied *β*-carboline alkaloid, was shown to block herpes simplex virus (HSV) infection by downregulating the NF-κB and mitogen-activated protein kinase pathways (9). 9N-methylharmine significantly reduced the dengue virus (DENV) titer (10). However, the effects of *β*-carboline alkaloids on PRV infection have not yet been elucidated.

Dual-specificity tyrosine phosphorylation–regulated kinases (DYRKs), which belong to the CMGC group of kinases, are considered key regulators of a wide range of cellular processes (11). Among the DYRK family, the most attractive target is DYRK1A because of its important role in the neuropathological traits of Down syndrome (12). DYRK1A, which is activated by the autophosphorylation of tyrosine residues in the activation loop, can phosphorylate its substrates on both serine and threonine residues and regulates the cell cycle, differentiation, and other biological processes (13). Inhibitors of DYRK1A, including harmine, were reported to exert strong antiviral effects against human cytomegalovirus (HCMV), varicella-zoster virus, and HSV (14). However, the role of DYRK1A in PRV proliferation remains unknown.

In the present study, a total of 107 *β*-carboline derivatives were screened for antiviral activity against PRV. Compound **45** was identified as the most effective inhibitor of PRV. Compound **45** significantly inhibited PRV proliferation both *in vitro* and *in vivo*. Furthermore, we provide evidence that **45** inhibits the macropinocytosis-dependent entry of PRV by targeting DYRK1A.

## Results

### Cytotoxic and antiviral activity of *β*-carboline derivatives *in vitro*

In order to investigate the cytotoxic effects of *β*-carbolines used in this study, we first evaluated cell viability using the Cell Counting Kit-8 assay. HeLa cells were treated with different *β*-carboline derivatives (5 μM). The results are shown in Figs. S1–S3. None of the compounds showed a cytotoxic effect at 5 μM. Thus, in the primary screening, a concentration of 5 μM was used for all *β*-carboline derivatives. The antiviral activities against PRV of the assayed *β*-carbolines were evaluated by plaque assay. Acyclovir (10 μM), which caused a reduction of 98%, was used as the positive control. Compared with dimethyl sulfoxide (DMSO)–treated cells, compounds that caused a reduction of ≥50% were considered active. In the primary screening, 20 *β*-carbolines were identified as potential inhibitors of PRV. Among them, nine compounds (**2**, **3**, **4**, **5**, **6**, **7**, **45**, **46**, and **48**) exhibited superior activity, with inhibitory rates of >90%. Four compounds (**3**, **45**, **46**, and **48**) showed excellent activity, with inhibitory rates of >99% (Figs. S1 and S2). Their antiviral effects were confirmed by quantitative PCR (qPCR) (Fig. S4). In addition, these compounds significantly inhibited the proliferation of Bartha-K61, suggesting that antiviral activities were not strain specific (Fig. S5).

**Figure 1:**
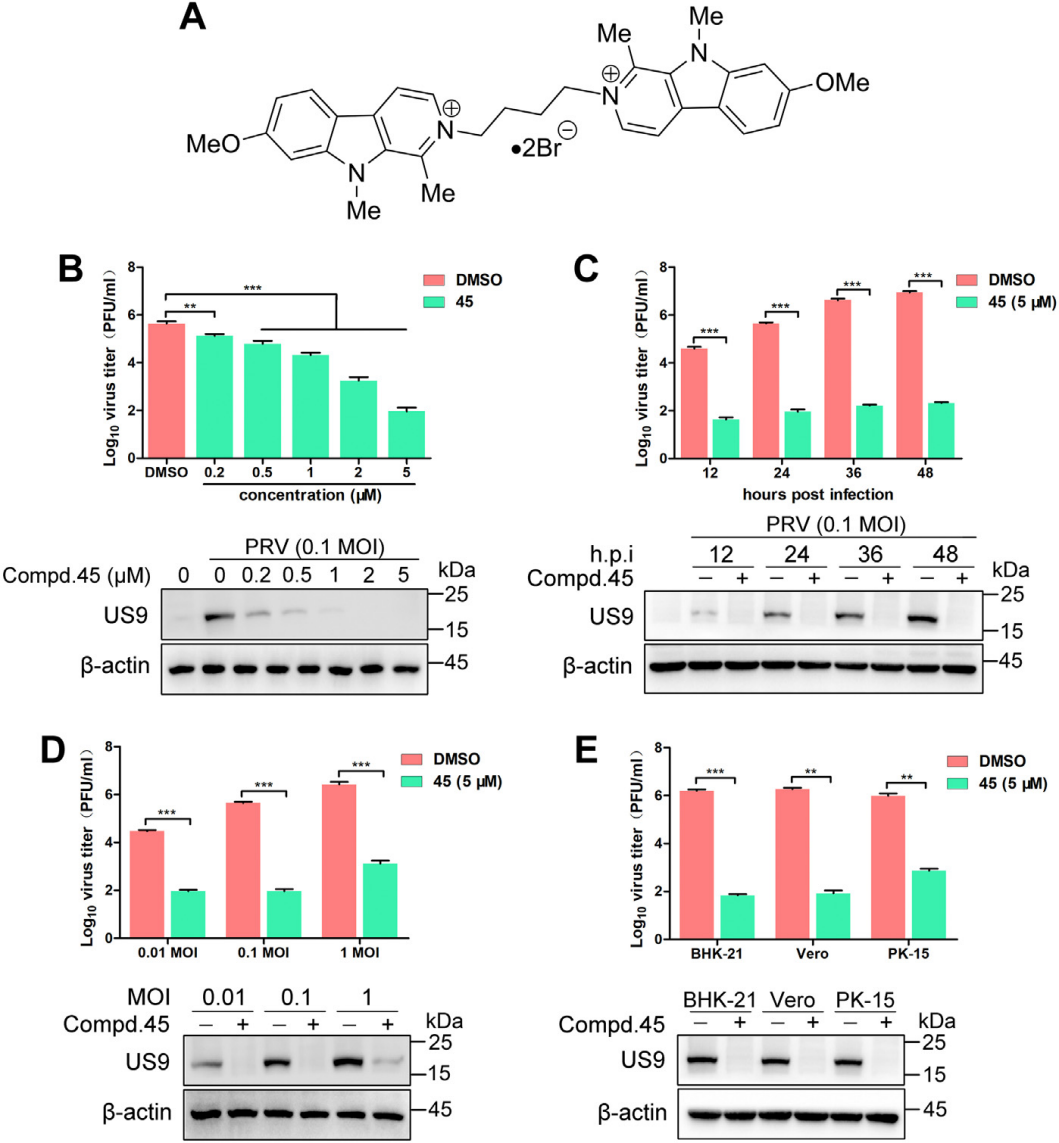
**Compound 45 inhibits PRV proliferation *in vitro*.***A*, structure of compound **45**. *B*, **45** inhibits PRV proliferation in a dose-dependent manner. HeLa cells were infected with PRV (0.1 MOI), and the medium was changed for fresh medium containing **45** at indicated concentrations, followed by a 24 h incubation. Viral titer was assessed by plaque assay (*top*). The viral protein was assessed by Western blot (*bottom*). Mean values ± SDs are shown (n = 3). Significance was assessed with two-tailed unpaired Student’s *t* test, ∗∗*p* < 0.01 and ∗∗∗*p* < 0.001. *C*, the antiviral effect of **45** against PRV at various time points. HeLa cells were infected with PRV (0.1 MOI), and the medium was changed for fresh medium containing **45** (5 μM). The antiviral effect of **45** at indicated time points was assessed by plaque assay (*top*) or Western blot (*bottom*). Mean values ± SDs are shown (n = 3). Significance was assessed with two-tailed unpaired Student’s *t* test, ∗∗∗*p* < 0.001. *D*, the antiviral effect of **45** against PRV (different MOIs). HeLa cells were infected with PRV (0.01, 0.1, or 1 MOI), and the medium was changed for fresh medium containing **45** (5 μM). The antiviral effect of **45** against PRV was assessed by plaque assay (*top*) or Western blot (*bottom*) at 24 h postinfection. Mean values ± SDs are shown (n = 3). Significance assessed with two-tailed unpaired Student’s *t* test, ∗∗∗*p* < 0.001. *E*, the antiviral effect of **45** in various cell lines. BHK-21, Vero, or PK-15 cells were infected with PRV (0.1 MOI), and the medium was changed for fresh medium containing **45** (5 μM). The antiviral effect of **45** in indicated cell lines was assessed by plaque assay (*top*) or Western blot (*bottom*) at 24 h postinfection. Mean values ± SDs are shown (n = 3). Significance was assessed with two-tailed unpaired Student’s *t* test, ∗∗*p* < 0.01 and ∗∗∗*p* < 0.001. MOI, multiplicity of infection, PRV, pseudorabies virus.

The CC_50_ and IC_50_ values were further determined by Cell Counting Kit-8 and plaque assay, respectively. Since the solubility of different *β*-carboline derivatives was variant, cell viability was determined in the range of 5 to 30 μM. All *β*-carboline derivatives displayed CC_50_ values higher than 30 μM. As shown in Table 1, 20 derivatives displayed anti-PRV activity, with IC_50_ values in the range of 0.032 to 4.08 μM. Compared with acyclovir, 14 derivatives showed superior activities, with IC_50_ values lower than 2.81 μM. The selectivity index (SI) was calculated as the CC_50_/IC_50_ ratio. In total, 16 derivatives emerged with SI ≥10. Among them, **45** showed the strongest antiviral activity, with the highest SI value (>937). Therefore, **45** was used in the following experiments. The structure of **45** is presented in Figure 1*A*. As shown in Figure 1*B*, treatment with **45** reduced the viral titer and viral protein load in a dose-dependent manner. In the presence of **45** (5 μM), the production of infectious virus particles was reduced by 3.7 Log, compared with the cells treated with DMSO. When the cells were treated with **45** (5 μM) for 12 to 48 h, the production of infectious virus particles and the viral protein load were both significantly reduced (Fig. 1*C*). With high doses of PRV infection (1 multiplicity of infection [MOI]), **45** also caused a significant reduction of 3.3 Log in the viral titer (Fig. 1*D*). As shown in Figure 1*E*, **45** caused reductions of 4.4 Log, 4.3 Log, and 3.1 Log in BHK-21, Vero, and PK-15, respectively. As shown in Table 2, the results revealed that **45** displayed anti-PRV activity with IC_50_ values in the range of 0.039 to 0.091 μM in these cell lines. Taken together, these results fully prove the anti-PRV effect of **45**.

**Table 1:** The anti-PRV activity of *β*-carboline derivatives in HeLa cells

Compound	CC_50_^a^ (μM)	IC_50_^b^ (μM)	SI^c^
**2**	>30	1.19 ± 0.09	>27
**3**	>30	0.52 ± 0.04	>60
**4**	>30	1.14 ± 0.12	>27
**5**	>30	1.56 ± 0.19	>20
**6**	>30	0.89 ± 0.07	>37
**7**	>30	1.31 ± 0.17	>22
**8**	>30	2.55 ± 0.38	>12
**9**	>30	1.95 ± 0.11	>15
**10**	>30	3.32 ± 0.33	>9
**11**	>30	2.59 ± 0.17	>11
**13**	>30	3.26 ± 0.12	>9
**14**	>30	2.11 ± 0.08	>14
**21**	>30	2.82 ± 0.14	>10
**45**	>30	0.032 ± 0.013	>937
**46**	>30	0.671 ± 0.059	>44
**48**	>30	0.128 ± 0.024	>234
**79**	>30	3.76 ± 0.06	>7
**80**	>30	2.46 ± 0.46	>12
**91**	>30	2.97 ± 0.44	>10
**107**	>30	4.08 ± 0.24	>7
**Acyclovir**	—d	2.81 ± 0.30	—d

The cell viability was determined by a CCK-8 kit, and CC_50_ values were calculated.

a HeLa cells were treated with different concentrations of each compound at 37 °C for 48 h.

b HeLa cells, infected with PRV (MOI = 0.1), were treated with different concentrations of each compound for 24 h. Supernatants were harvested to determine the virus production by a plaque assay. IC_50_ values were calculated (mean ± SD).

c Selectivity index; SI = CC_50_/IC_50_.

d Not examined.

**Table 2:** The anti-PRV activity of compound **45** in BHK-21, PK-15, and Vero cells

Compound	Cell line	CC_50_^a^ (μM)	IC_50_^b^ (μM)	SI^c^
**45**	BHK-21	>30	0.039 ± 0.003	>769
	PK-15	>30	0.091 ± 0.09	>329
	Vero	>30	0.052 ± 0.021	>576

a BHK-21, PK-15, or Vero cells were treated with different concentrations of each compound at 37 °C for 48 h. The cell viability was determined by a CCK-8 kit, and CC_50_ values were calculated.

b BHK-21, PK-15, or Vero cells were infected with PRV (MOI = 0.1) and then treated with different concentrations of each compound for 24 h. Supernatants were harvested to determine the virus production by a plaque assay. IC_50_ values were calculated (mean ± SD).

c Selectivity index; SI = CC_50_/IC_50_.

### Mode of antiviral action of 45 against PRV

**Figure 2:**
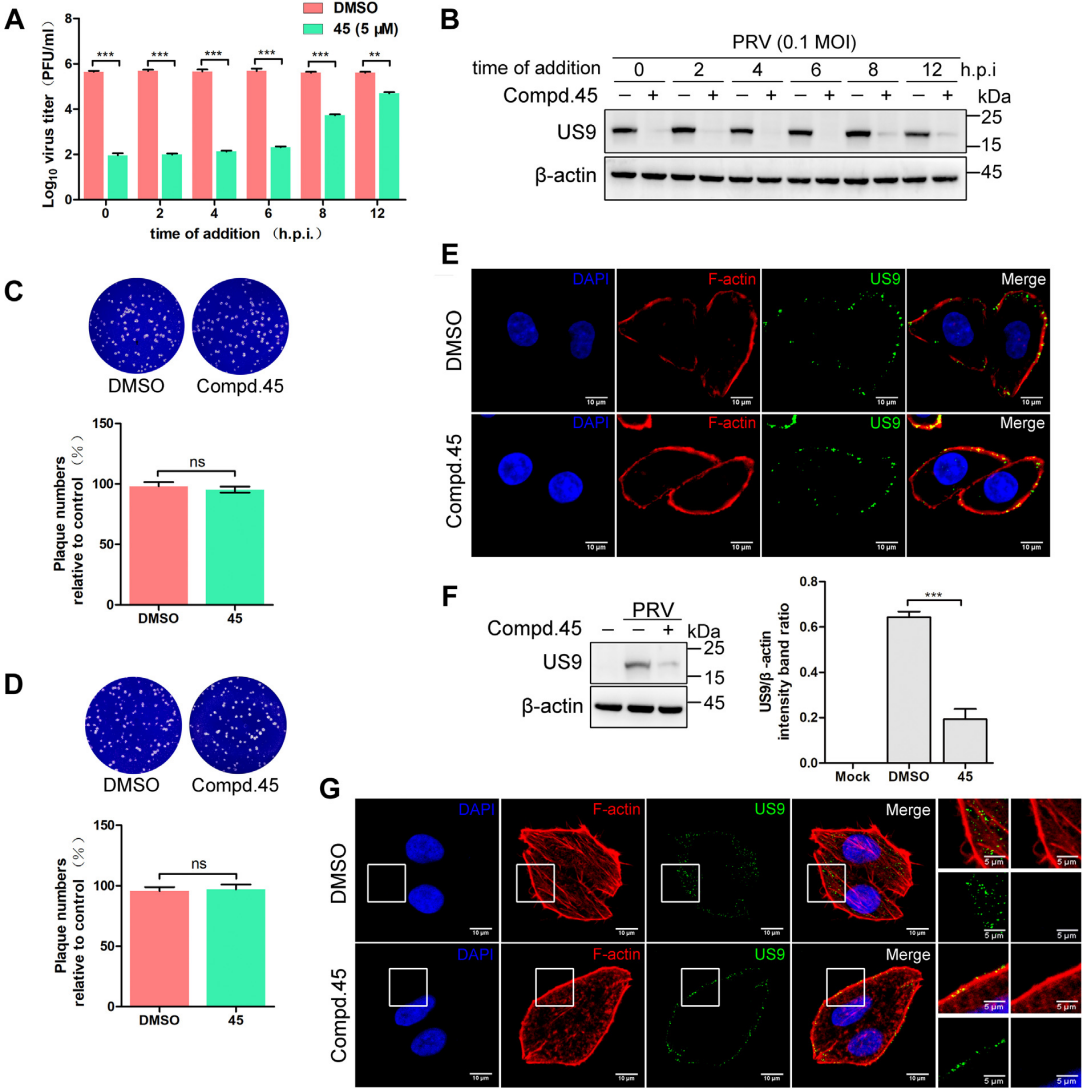
**Compound 45 inhibits PRV entry but not adsorption.***A* and *B*, the antiviral effect of adding **45** at various time points. HeLa cells were infected with PRV (0.1 MOI). **45** (5 μM) was added at the indicated time points postinfection, and the antiviral effect was detected by plaque assay (*A*) or Western blot (*B*) at 24 h postinfection. Mean values ± SDs are shown (n = 3). Significance was assessed with two-tailed unpaired Student’s *t* test, ∗∗*p* < 0.01 and ∗∗∗*p* < 0.001. *C*, **45** has no virucidal effect on PRV. HeLa cells were infected with pretreated PRV. After 1 h of adsorption, cells were washed three times with PBS and covered with medium-containing methylcellulose (1%). When the plaques were visible, cells were fixed and stained with crystal violet. Mean values ± SDs are shown (n = 3). Significance was assessed with two-tailed unpaired Student’s *t* test; ns, not significant. *D*, **45** has no effect on PRV adsorption. HeLa cells were pretreated with **45** (5 μM) or DMSO for 1 h, prechilled at 4 °C for 1 h, infected with PRV (150 PFU) at 4 °C for 1 h, washed with PBS, and then covered with medium-containing methylcellulose (1%). When the plaques were visible, cells were fixed and stained with crystal violet. Mean values ± SDs are shown (n = 3). Significance was assessed with two-tailed unpaired Student’s *t* test; ns, not significant. *E*, **45** has no effect on PRV adsorption. HeLa cells were pretreated with **45** (5 μM) or DMSO for 1 h, prechilled at 4 °C for 1 h, infected with PRV (50 MOI) at 4 °C for 1 h, and washed with PBS. The adsorption of PRV was analyzed by indirect immunofluorescence. *F*, **45** inhibits PRV entry into HeLa cells. HeLa cells were treated with **45** (5 μM) at 37 °C for 1 h, prechilled at 4 °C for 1 h, and then infected with PRV (50 MOI) at 4 °C for 1 h. After that, cells were washed with a low-pH buffer and transferred into the incubator. After 4 h, the viral protein was measured by Western blot (*left*). The US9 protein levels relative to β-actin levels were determined by densitometry (*right*). Mean values ± SDs are shown (n = 3). Significance was assessed with two-tailed unpaired Student’s *t* test, ∗∗∗*p* < 0.001. *G*, **45** inhibits PRV entry into HeLa cells. HeLa cells were treated with **45** (5 μM) at 37 °C for 1 h, prechilled at 4 °C for 1 h, then infected with PRV (50 MOI) at 4 °C for 1 h. After that, cells were washed with prechilled PBS and transferred into the incubator to allow viral entry. After 1 h, cells were fixed and analyzed by indirect immunofluorescence. DMSO, dimethyl sulfoxide; MOI, multiplicity of infection; PRV, pseudorabies virus.

To further dissect which stage is targeted by **45** during PRV infection, a series of experiments were conducted, including a time of addition assay, a virucidal assay, an adsorption assay, and an entry assay. As shown in Figure 2*A*, the postinfection addition of **45** at 2, 4, or 6 h significantly reduced the viral titers compared with the control group. When **45** was added at 8 h or 12 h postinfection, its antiviral effect was weakened. Similar results were obtained by Western blot analysis; when **45** was added at the early time points, the viral protein was almost undetectable (Fig. 2*B*). These results suggest that **45** may affect the early stage of the PRV life cycle. To determine whether **45** has a virucidal effect on PRV, PRV was incubated with a high concentration of **45** (50 μM) for 2 h, and infectious viral particles were detected by plaque assay. As shown in Figure 2*C*, treatment with **45** had no direct toxic effect on viral particles. US9, a transmembrane protein in the viral envelope, could be used as a marker for virus adsorption. As shown in Figure 2, *D* and *E*, the viral particles binding to cells were not affected by **45** treatment. Then the effect of **45** on PRV entry was detected by Western blot and indirect immunofluorescence. As shown in Figure 2*F*, treatment with **45** significantly reduced the viral protein load at 4 h postinfection (0.64 ± 0.02 and 0.19 ± 0.03 for DMSO and **45** treatment samples, respectively). Moreover, treatment with **45** could efficiently block PRV uptake at 1 h postinfection (Fig. 2*G*). These results prove that **45** blocks the entry of PRV into HeLa cells.

### Compound **45** inhibits macropinocytosis in HeLa cells

**Figure 3:**
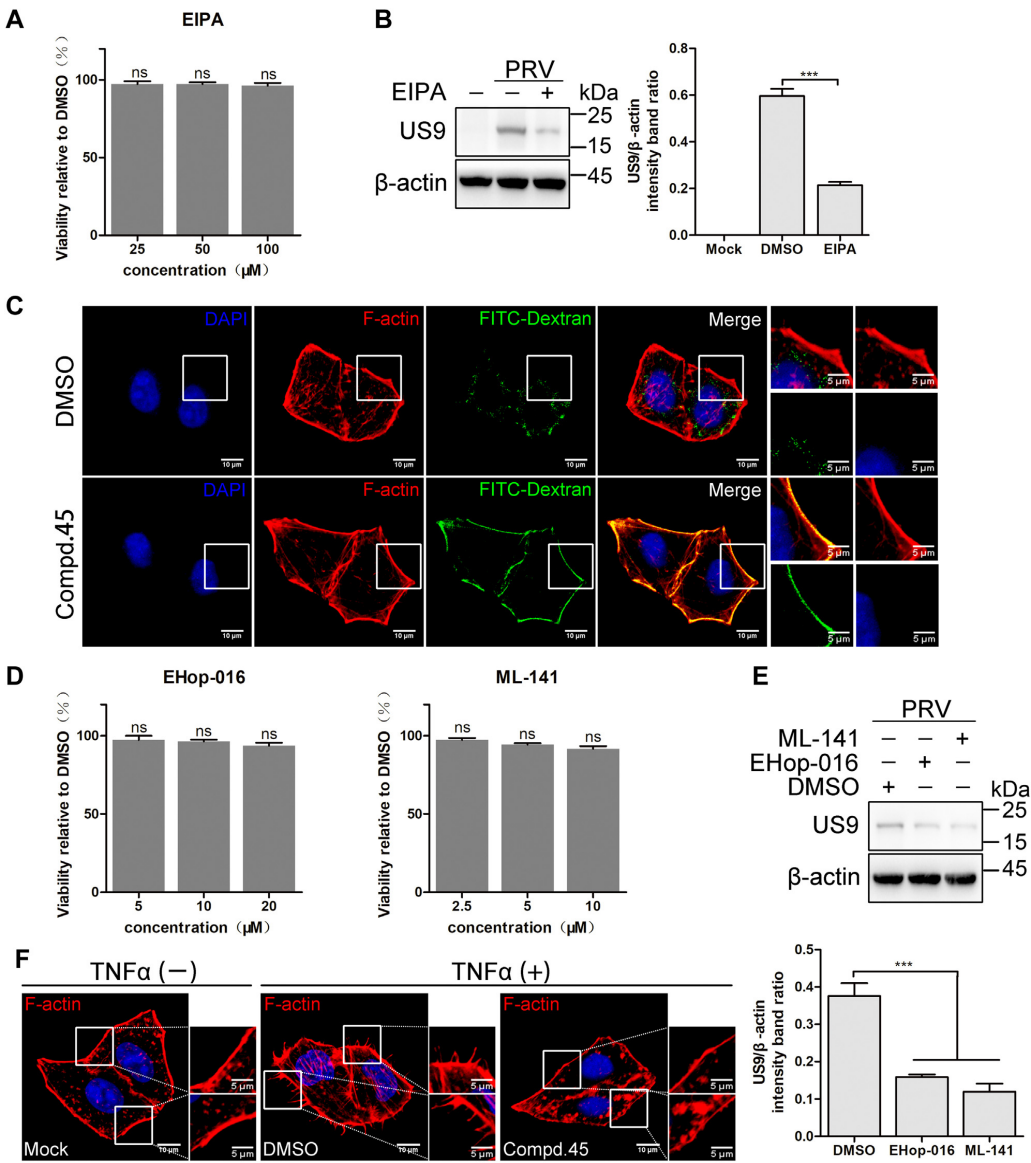
**Compound 45 inhibits macropinocytosis in HeLa cells.***A*, cell viability of DF-1 cells treated with EIPA. DF-1 cells were incubated with indicated concentrations of EIPA. At 48 h postincubation, the cell viability was determined using a CCK-8 kit. Mean values ± SDs are shown (n = 3). Significance was assessed with two-tailed unpaired Student’s *t* test; ns, not significant. *B*, EIPA inhibits PRV entry into HeLa cells. HeLa cells were treated with EIPA (50 μM) at 37 °C for 1 h, prechilled at 4 °C for 1 h, and then infected with PRV (50 MOI) at 4 °C for 1 h. After that, cells were washed with a low-pH buffer and transferred into the incubator. After 4 h, the viral protein was measured by Western blot (*left*). The US9 protein levels relative to β-actin levels were determined by densitometry (*right*). Mean values ± SDs are shown (n = 3). Significance was assessed with two-tailed unpaired Student’s *t* test. ∗∗∗*p* < 0.001. *C*, **45** inhibits the FITC–dextran uptake. HeLa cells were treated with **45** (5 μM) at 37 °C for 1.5 h, incubated with FITC–dextran 70K (5 mg/ml) for 30 min, and washed. After that, cells were fixed, and the FITC–dextran uptake was analyzed by indirect immunofluorescence. *D*, cell viability of DF-1 cells treated with EHop-016 or ML-141. DF-1 cells were incubated with indicated concentrations of EHop-016 (*left*) or ML-141 (*right*). At 48 h postincubation, the cell viability was determined using a CCK-8 kit. Mean values ± SDs are shown (n = 3). Significance was assessed with two-tailed unpaired Student’s *t* test; ns, not significant. *E*, EHop-016 and ML-141 inhibit PRV entry into HeLa cells. HeLa cells were treated with EHop-016 (10 μM) or ML-141 (5 μM) at 37 °C for 1 h, prechilled at 4 °C for 1 h, and then infected with PRV (50 MOI) at 4 °C for 1 h. After that, cells were washed with a low-pH buffer and transferred into the incubator. After 4 h, the viral protein was measured by Western blot (*upper*). The US9 protein levels relative to β-actin levels were determined by densitometry (*lower*). Mean values ± SDs are shown (n = 3). Significance was assessed with two-tailed unpaired Student’s *t* test: ∗∗∗*p* < 0.001. *F*, **45** inhibits the membrane protrusion induced by TNFα in HeLa cells. Serum-starved HeLa cells were treated with **45** (5 μM) or DMSO for 1 h and then challenged with TNFα (100 ng/ml) for 10 min. After that, cells were fixed and processed for indirect immunofluorescence assay. CCK-8, Cell Counting Kit-8; DMSO, dimethyl sulfoxide; EIPA, 5-[*N*-ethyl-*N*-isopropyl] amiloride; MOI, multiplicity of infection; PRV, pseudorabies virus; TNFα, tumor necrosis factor alpha.

Lv *et al.* (15) found that macropinocytosis is the major pathway of PRV entry into HeLa cells. To confirm this, HeLa cells were pretreated with 5-[*N*-ethyl-*N*-isopropyl] amiloride (EIPA; an inhibitor of macropinocytosis) at a concentration without cytotoxicity (Fig. 3*A*). As shown in Figure 3*B*, EIPA treatment significantly reduced the viral protein load at 4 h postinfection (0.59 ± 0.02 and 0.21 ± 0.01 for DMSO and EIPA treatment samples, respectively). Dextran is a well-known fluid-phase marker for macropinosomes. Therefore, to assess the effect of **45** on macropinocytosis, an FITC–dextran uptake assay was conducted. HeLa cells were pretreated with **45** (5 μM) for 1.5 h and then incubated with FITC–dextran for 1 h. As indicated in Figure 3*C*, treatment with **45** markedly decreased FITC–dextran uptake. It has been reported that macropinocytosis relies on the formation of elaborate membrane protrusions, which can be regulated by Rac1 and Cdc42 (16). Lv *et al.* (15) found that inhibition of the function of Rac1 and Cdc42 significantly decreased the internalization of PRV in HeLa cells. To confirm this, HeLa cells were pretreated with EHop-016 (an inhibitor of Rac1) or ML-141 (an inhibitor of Cdc42) at concentrations without cytotoxicity (Fig. 3*D*). As expected, treatment with EHop-016 and ML-141 both reduced the viral protein load (Fig. 3*E*). Previous reports showed that tumor necrosis factor alpha (TNFα) could induce the formation of protrusions through the activation of Cdc42 (17). As shown in Figure 3*F*, treatment with TNFα (100 ng/ml) promoted the formation of protrusions on the surface of cells, whereas **45** significantly inhibited the effects of TNFα. Together, these results suggest that **45** blocks macropinocytosis and the formation of membrane protrusions in HeLa cells.

### Inhibition of DYRK1A blocks the macropinocytosis-dependent entry of PRV

**Figure 4:**
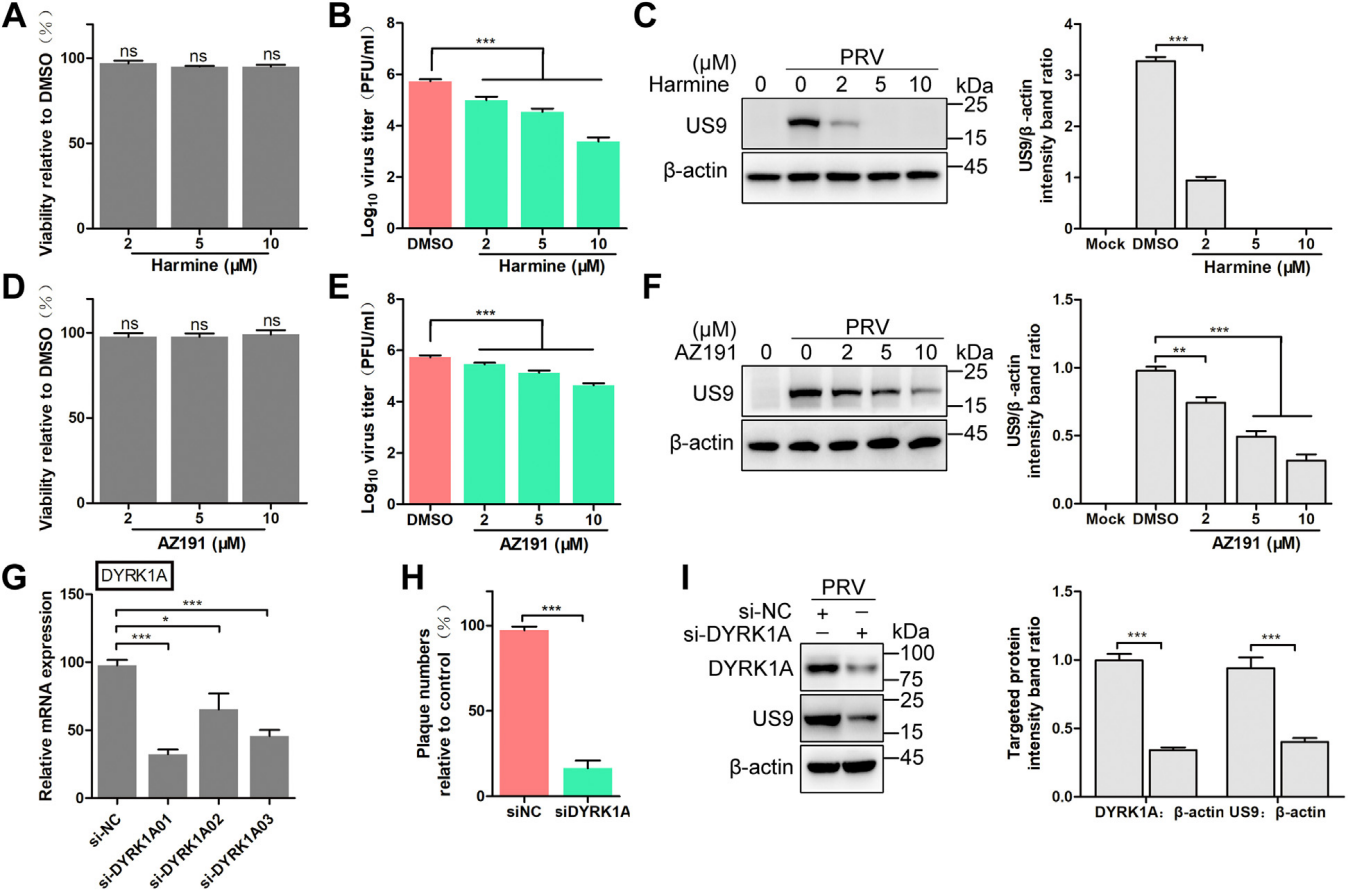
**Inhibition of DYRK1A suppresses PRV proliferation in HeLa cells.***A*, cell viability of DF-1 cells treated with harmine. DF-1 cells were incubated with indicated concentrations of harmine. At 48 h postincubation, the cell viability was determined using a CCK-8 kit. Mean values ± SDs are shown (n = 3). Significance was assessed with two-tailed and unpaired Student’s *t* test; ns, not significant. *B* and *C*, harmine inhibits PRV proliferation in a dose-dependent manner. HeLa cells were infected with PRV (0.1 MOI), and the medium was changed for fresh medium containing harmine at indicated concentrations, followed by a 24 h incubation. Viral titer was assessed by plaque assay (*B*). The viral protein was assessed by Western blot (*C*, *left*). The US9 protein levels relative to β-actin levels were determined by densitometry (*C*, *right*). Mean values ± SDs are shown (n = 3). Significance was assessed with two-tailed unpaired Student’s *t* test: ∗∗∗*p* < 0.001. *D*, cell viability of DF-1 cells treated with AZ191. DF-1 cells were incubated with indicated concentrations of AZ191. At 48 h postincubation, the cell viability was determined using a CCK-8 kit. Mean values ± SDs are shown (n = 3). Significance was assessed with two-tailed and unpaired Student’s *t* test; ns, not significant. *E* and *F*, AZ191 inhibits PRV proliferation in a dose-dependent manner. HeLa cells were infected with PRV (0.1 MOI), and the medium was changed for fresh medium containing AZ191 at indicated concentrations, followed by a 24 h incubation. Viral titer was assessed by plaque assay (*E*). The viral protein was assessed by Western blot (*F*, *left*). The US9 protein levels relative to β-actin levels were determined by densitometry (*F*, *right*). Mean values ± SDs are shown (n = 3). Significance was assessed with two-tailed and unpaired Student’s *t* test. ∗∗*p* < 0.01, ∗∗∗*p* < 0.001. *G*, knockdown efficiency of siRNAs targeting DYRK1A. HeLa cells were transfected with siRNAs targeting DYRK1A. At 24 h post-transfection, cells were harvested to assess the knockdown efficiency by qPCR. Mean values ± SDs are shown (n = 4). Significance was assessed with two-tailed and unpaired Student’s *t* test: ∗*p* < 0.05 and ∗∗∗*p* < 0.001. *H* and *I*, knockdown of DYRK1A inhibits PRV proliferation. HeLa cells were transfected with si-DYRK1A01. At 24 h post-transfection, cells were infected with PRV (0.1 MOI). At 24 h postinfection, the supernatant was harvested to assess the viral titer by plaque assay (*H*). Cells were harvested to assess the expression of viral protein by Western blot (*I*, *left*). The targeted protein levels relative to β-actin levels were determined by densitometry (*I*, *right*). Mean values ± SDs are shown (n = 3). Significance was assessed with two-tailed and unpaired Student’s *t* test: ∗∗∗*p* < 0.001. CCK-8, Cell Counting Kit-8; DYRK1A, dual-specificity tyrosine phosphorylation–regulated kinase 1A; MOI, multiplicity of infection; PRV, pseudorabies virus; qPCR, quantitative PCR.

To determine the most probable targets of **45**, the structure of **45** was uploaded into Swiss Target Prediction (http://www.swisstargetprediction.ch/) (18). The top 10 predicted targets of **45** are presented in Table 3. Four predicted targets belong to the family of DYRKs, including DYRK2, DYRK4, DYRK1A, and DYRK3. Hutterer *et al.*(14) found that inhibitors of DYRK1A and DYRK1B displayed superior antiviral activity against HCMV, compared with inhibitors of DYRK2, DYRK3, and DYRK4. Thus, based on the predicted targets and the literature, we determined the effect of DYRK1 on the proliferation of PRV. Harmine (compound **2**), a known DYRK1A inhibitor, could significantly suppress the production of infectious virus particles and viral protein expression without cytotoxicity (Fig. 4, *A*–*C*). In the presence of 10 μM harmine, the production of infectious virus particles was reduced by 2.3 Log, compared with the cells treated with DMSO. The role of DYRK1B, the most closely related kinase to DYRK1A, in PRV infection is unknown. To verify the effect of DYRK1B, AZ191, an inhibitor of DYRK1B, was used in this study. At a concentration of 10 μM, AZ191 did not affect cell viability (Fig. 4*D*). The specificity of AZ191 for DYRK1B is about fivefold greater than that for DYRK1A. Treatment with AZ191 also reduced the viral titer and the viral protein load in a dose-dependent manner. In the presence of 10 μM AZ191, the production of infectious virus particles was reduced by 1.1 Log (Fig. 4, *E* and *F*). These results suggest that inhibition of DYRK1 suppresses PRV proliferation, whereas inhibition of DYRK1A exhibits stronger anti-PRV activity. To confirm the role of DYRK1A during PRV infection of cells, DYRK1A was knocked down in HeLa cells using specific siRNAs. The knockdown efficiency of different siRNAs was determined by qPCR, and the most efficient siRNA, si-DYRK1A01, was selected for subsequent assays (Fig. 4*G*). As shown in Figure 4, *H* and *I*, the viral titer and viral protein load decreased after knockdown of DYRK1A. These results demonstrate the importance of DYRK1A for PRV proliferation.

**Table 3:** Top 10 predicted targets of compound **45**

Target	Common name	UniProt ID	Target class
Serine/threonine-protein kinase haspin	HASPIN	Q8TF76	Kinase
Dual-specificity tyrosine-phosphorylation regulated kinase 2	DYRK2	Q92630	Kinase
Butyrylcholinesterase	BCHE	P06276	Hydrolase
Acetylcholinesterase	ACHE	P22303	Hydrolase
Dual specificity tyrosine-phosphorylation-regulated kinase 4	DYRK4	Q9NR20	Kinase
Dual-specificity tyrosine-phosphorylation regulated kinase 1A	DYRK1A	Q13627	Kinase
Nischarin	NISCH	Q9Y2I1	Other cytosolic proteins
Dual-specificity tyrosine-phosphorylation regulated kinase 3	DYRK3	O43781	Kinase
Monoamine oxidase A	MAOA	P21397	Oxidoreductase
Monoamine oxidase B	MAOB	P27338	Oxidoreductase

**Figure 5:**
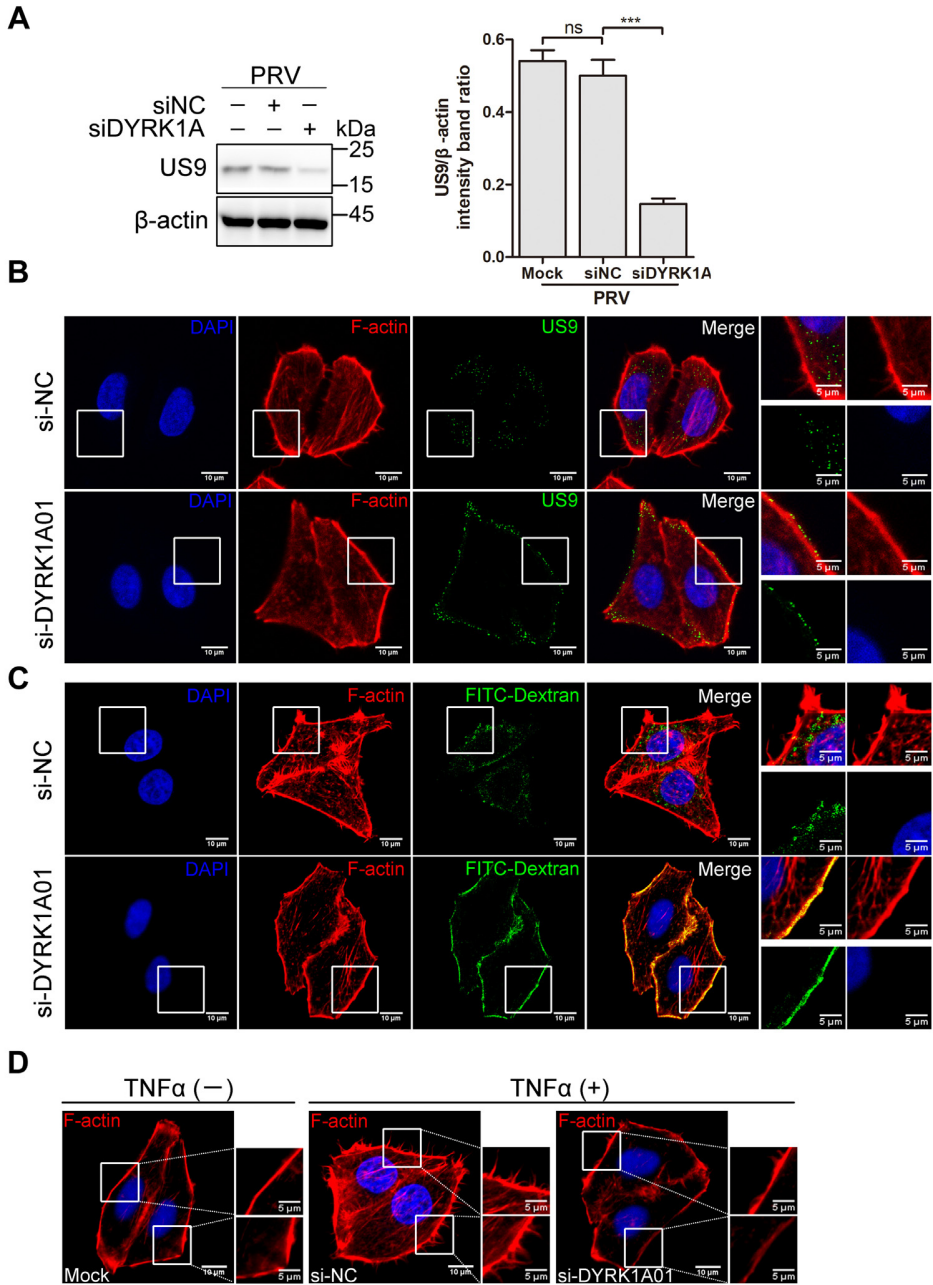
**Knockdown of DYRK1A inhibits PRV entry and macropinocytosis in HeLa cells.***A*, knockdown of DYRK1A inhibits PRV entry into HeLa cells. HeLa cells were transfected with si-DYRK1A01. At 24 h post-transfection, cells were prechilled at 4 °C for 1 h and then infected with PRV (50 MOI) at 4 °C for 1 h. After that, cells were washed with a low pH buffer and transferred into the incubator. The viral protein was measured by Western blot at 4 h postinfection (*left*). The US9 protein levels relative to β-actin levels were determined by densitometry (*right*). Mean values ± SDs are shown (n = 3). Significance was assessed with two-tailed and unpaired Student’s *t* test; ns, not significant, ∗∗∗*p* < 0.001. *B*, knockdown of DYRK1A inhibits PRV entry into HeLa cells. HeLa cells were transfected with si-DYRK1A01. At 24 h post-transfection, cells were prechilled at 4 °C for 1 h and then infected with PRV (50 MOI) at 4 °C for 1 h. After that, cells were washed with prechilled PBS and transferred into the incubator. At 1 h postinfection, the entry of PRV was analyzed by indirect immunofluorescence. *C*, knockdown of DYRK1A inhibits the FITC–dextran uptake. HeLa cells were transfected with si-DYRK1A01. At 24 h post-transfection, cells were incubated with FITC–dextran 70K (5 mg/ml) for 1 h and washed. After that, cells were fixed, and the FITC–dextran uptake was analyzed by indirect immunofluorescence. *D*, knockdown of DYRK1A inhibits the membrane protrusions induced by TNFα in HeLa cells. HeLa cells were transfected with si-DYRK1A01. At 24 h post-transfection, cells were serum-deprived for 10 h and then challenged with TNFα (100 ng/ml) for 10 min. After that, cells were fixed and processed for indirect immunofluorescence. DYRK1A, dual-specificity tyrosine phosphorylation–regulated kinase 1A; MOI, multiplicity of infection; PRV, pseudorabies virus; TNFα, tumor necrosis factor alpha.

The effect of DYRK1A knockdown on PRV entry was detected by Western blot and indirect immunofluorescence. As shown in Figure 5*A*, knockdown of DYRK1A significantly reduced the viral protein load at 4 h postinfection (0.50 ± 0.04 and 0.15 ± 0.01 for mock samples and DYRK1A knockdown samples, respectively). Moreover, the results of indirect immunofluorescence assays also demonstrated that DYRK1A knockdown efficiently blocked PRV entry (Fig. 5*B*). To evaluate whether DYRK1A knockdown affects macropinocytosis, an FIT–dextran uptake assay was conducted. As indicated in Figure 5*C*, DYRK1A knockdown markedly decreased FITC–dextran uptake. The TNFα-induced formation of membrane protrusions was attenuated by DYRK1A knockdown (Fig. 5*D*). Together, these results suggest that inhibition of DYRK1A could inhibit the macropinocytosis-dependent entry of PRV.

### Validation of DYRK1A as a direct target of **45**

**Figure 6:**
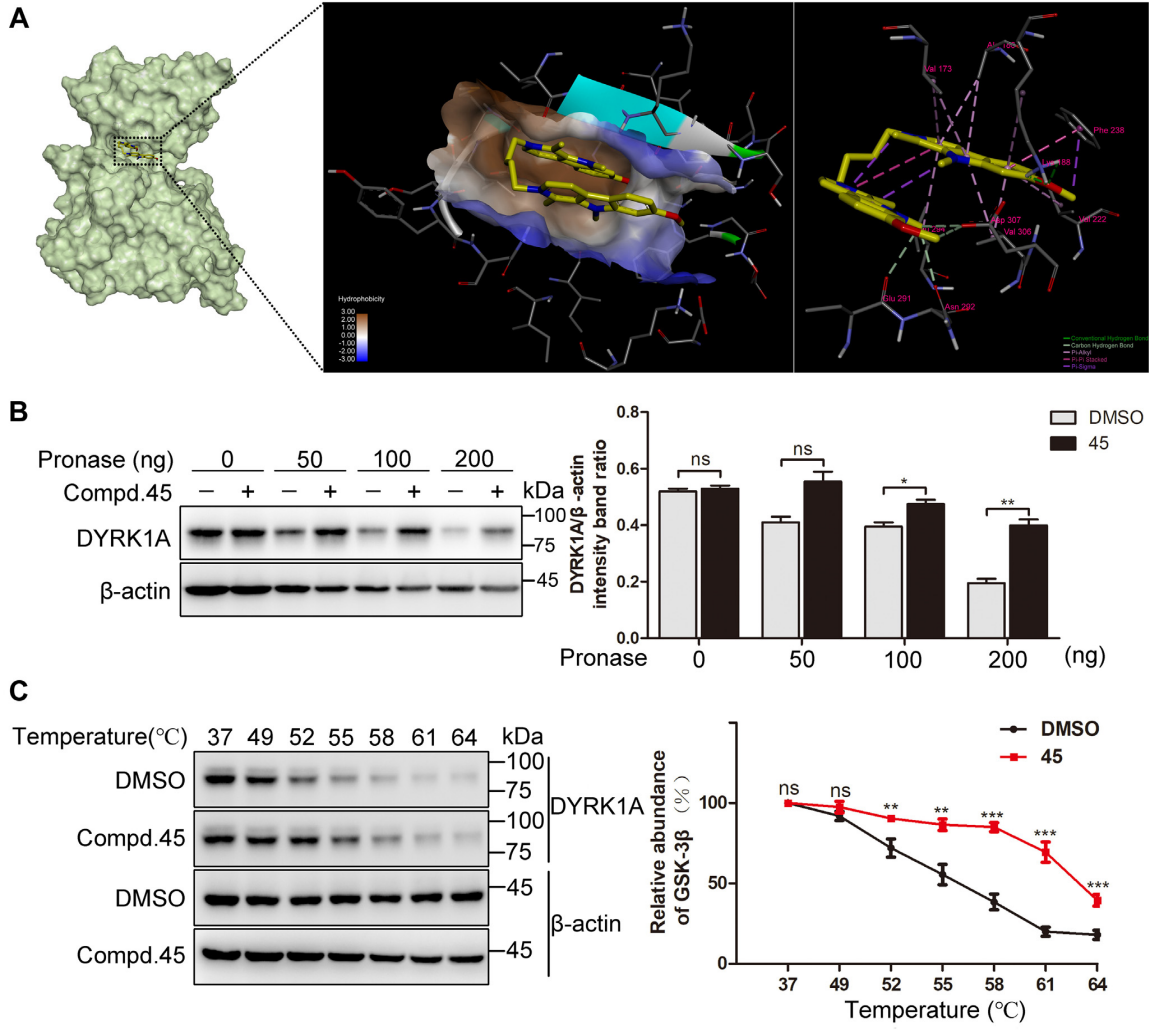
**Direct binding of 45 to the DYRK1A.***A*, molecular docking of simulation of the binding of **45** to DYRK1A. *B*, **45** protects DYRK1A against proteolysis. HeLa cells were harvested and treated with DMSO or **45** (100 μM). The stability of DYRK1A against proteolysis was assessed by DARTS (*left*). The relative quantification of the target protein level was analyzed by densitometry (*right*). Mean values ± SDs are shown (n = 3). Significance was assessed with two-tailed and unpaired Student’s *t* test; ns, not significant, ∗*p* < 0.05, ∗∗*p* < 0.01. *C*, **45** protects DYRK1A from degradation because of heating. HeLa cells were harvested and treated with DMSO or **45** (100 μM). The thermal stability of DYRK1A was assessed by CETSA (*left*). The relative quantification of the target protein level was analyzed by densitometry (*right*). Mean values ± SDs are shown (n = 3). Significance was ssessed with two-tailed and unpaired Student’s *t* test; ns, not significant, ∗∗*p* < 0.01, ∗∗∗*p* < 0.001. CETSA, cellular thermal shift assay; DARTS, drug affinity responsive target stability; DMSO, dimethyl sulfoxide; DYRK1A, dual-specificity tyrosine phosphorylation–regulated kinase 1A.

Molecular docking was conducted to predict the potential interaction between DYRK1A and **45** by Autodock 4 and Autodock Vina (The Scripps Research Institute). The predicted inhibitory constant (Ki) was 15.8 nM. The binding energy was predicted to be −10.64 kcal/mol. As shown in Figure 6*A*, **45** is predicted to dock into the ATP-binding pocket of DYRK1A where it could form hydrophobic contacts with Val173, Ala186, Lys188, Val222, Phe238, Leu294, and Val306. Compound **45** is predicted to form two hydrogen bonds with Lys188; these hydrogen bond distances were calculated to be 2.03 and 2.21 Å. Moreover, **45** is predicted to interact with Glu291, Asn292, and Asp307 through carbon–hydrogen bonds (nonclassical hydrogen bonds). To further characterize the binding of **45** to DYRK1A, drug affinity responsive target stability (DARTS) and cellular thermal shift assay (CETSA) analyses were conducted. DARTS is a label-free method based on the principle that binding of a molecule to a target protein could stabilize the target protein by increasing its resistance to proteases (19). HeLa cell lysates were treated with DMSO or **45** at 4 °C overnight, and the lysates were then exposed to increasing doses of pronase. As expected, **45** protected DYRK1A against pronase digestion. In the presence of 200 ng pronase, **45** significantly increased the amounts of DYRK1A (0.19 ± 0.01 and 0.40 ± 0.02 for DMSO and **45** treatment samples, respectively). Compound **45** did not prevent pronase digestion of β-actin, which was used as a control protein (Fig. 6*B*). CETSA is based on the principle that the engagement of a ligand with a protein could change the thermal stability of the target protein (20). HeLa cell lysates were incubated with DMSO or **45** and heated at the indicated temperatures. As shown in Figure 6*C*, **45** increased the amounts of DYRK1A under heating at 58 °C (0.38 ± 0.04 and 0.85 ± 0.02 for DMSO and **45** treatment samples, respectively). Compound **45** changed the melting curve of DYRK1A compared with DMSO, suggesting the occurrence of ligand-induced stabilization. In combination, these results illustrate that **45** directly interacts with DYRK1A.

### *In vivo* antiviral efficacy of **45** against PRV

To estimate the *in vivo* toxicity of **45**, mice were intraperitoneally injected with **45** (1 mg/kg or 10 mg/kg). At 14 days postinjection, no death occurred, and there was no abnormality in body weight. The weights of the liver, spleen, and kidney were also unchanged, as shown in Table 4. These results indicate that **45** is safe to use at the doses used in this study.

**Table 4:** Animal grouping and the weight of body and organs

Group (numbers)	Compound **45**	Weight (g)	Liver	Spleen	Kidney
Group I (10)	—	33.86 ± 1.88	4.63 ± 0.12	0.35 ± 0.11	1.44 ± 0.11
Group II (10)	1 mg/kg	33.50 ± 3.81	4.98 ± 0.20	0.36 ± 0.08	1.48 ± 0.24
Group III (10)	10 mg/kg	34.26 ± 1.20	4.79 ± 0.45	0.32 ± 0.13	1.63 ± 0.15

**Figure 7:**
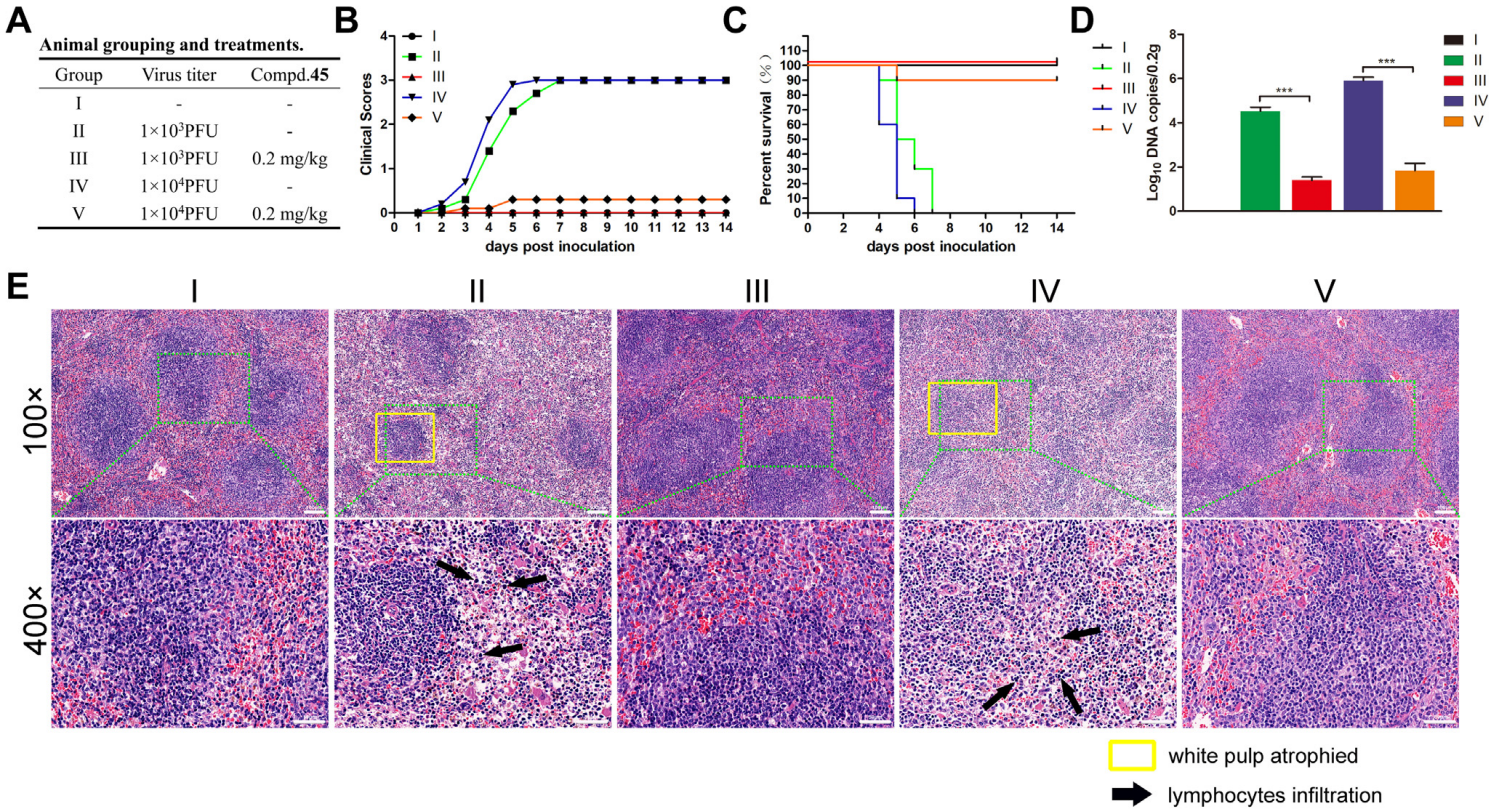
**45 inhibits PRV proliferation *in vivo*.***A*, animal grouping and treatments. *B*, **45** decreases the clinical scores compared with the control group. The average daily clinical scores of the mice from each group were calculated throughout the experimental period. *C*, **45** protects mice from PRV-induced death. The percent survival (%) in each group was calculated and presented. *D*, **45** decreases the viral DNA copies in spleens. The spleens were collected at 72 h postinoculation, and the viral DNA copies were detected by qPCR. Mean values ± SDs are shown (n = 5). Significance was assessed with two-tailed and unpaired Student’s *t* test, ∗∗∗*p* < 0.001. *E*, **45** decreases the PRV-induced lesions in spleens. The spleens were collected at 72 h postinoculation and stained with hematoxylin and eosin. PRV, pseudorabies virus; qPCR, quantitative PCR.

To assess the *in vivo* antiviral activity of **45**, mice were intraperitoneally inoculated with PRV (1 × 10^3^ plaque-forming unit [PFU] or 1 × 10^4^ PFU) with or without **45** (0.2 mg/kg) (Fig. 7*A*). The state of the mice was recorded every 12 h. At 3 days after inoculation, mice of group II and group IV developed neurological symptoms of abnormal excitation, scratching, turning around, and ultimately death. The clinical scores are presented in Figure 7*B*. The mouse survival rate was recorded for 14 days. All mice in the challenge control groups (groups II and IV) were dead within 7 days. Treatment with **45** provided 100% and 90% protection in group III and V, respectively (Fig. 7*C*). To determine the viral DNA copies in the spleens of infected mice, five mice from each group were randomly selected at 72 h after inoculation, and total DNA was extracted from the spleen tissues of these mice. Viral DNA copies were detected using qPCR. As shown in Figure 7*D*, the viral DNA copies were significantly reduced after treatment with **45**. To further characterize the preventive effect of **45** on PRV infection, the spleens were subjected to histopathological examination. Upon PRV infection, atrophy of white pulp (yellow box) was observed, especially in group IV. The boundary between red pulp and white pulp turned indistinct in group IV but not in group V. In addition, these results revealed the diffuse infiltration of lymphocytes in red pulp (*black arrow*). The structure of red pulp also became loose. As expected, treatment with **45** could prevent these specific symptoms (Fig. 7*E*).

## Discussion

In 1984, a series of eudistomins that exerted modest antiviral activity against HSV-1 were isolated from Caribbean tunicate (*Eudistoma olivaceum*) (21). Since then, a large number of *β*-carboline derivatives have been reported to possess antiviral activity. Mazamine A, 8-hydroxymanzamine A, manzamine A monohydrochloride, and manzamine A monotartrate displayed excellent antiviral activity against HSV-1, with IC_50_ values of 1.0, 3.7, 0.1, and 0.1 μM, respectively (22). Harmine, originally isolated from *Peganum harmala* seeds, exhibited antiviral activity against HSV-1 (IC_50_ = 4.56 μM), HSV-2 (IC_50_ = 1.47 μM), enterovirus 71 (IC_50_ = 10 ± 2.50 μM), and HCMV (IC_50_ = 0.71 ± 0.019 μM) (9, 14, 23). Recently, we found that several C ring-truncated canthin-6-one analogs and 1-formyl-*β*-carboline derivatives could block the proliferation of Newcastle disease virus, which belongs to the paramyxoviruses (24, 25). However, to the best of our knowledge, the potential of *β*-carboline derivatives as antiviral agents against PRV has not been studied. In the present study, we found that 20 *β*-carboline derivatives inhibit PRV proliferation. Among them, **3** (9-methyl-harmine) displayed stronger anti-PRV activity than **2** (harmine). These data indicate that introduction of a methyl group at position-N^9^ helps to improve the anti-PRV activity. These data were consistent with the anti-DENV activity of 9-methyl-harmine and harmine (10). Moreover, when the C^7^-methoxy group (compound **3**) was replaced with a hydroxyl group (compound **8**), anti-PRV activity was decreased. The primary screening also showed that the O^7^-substituted dimers and N^9^-substituted dimers had no antiviral effect on PRV. These results highlight the importance of substituent groups at positions O^7^ and N^9^. In 2020, four heterodimers consisting of tetracyclic *β*-carboline and tricyclic *β*-carboline alkaloids were isolated from the seeds of *P. harmala*. These heterodimers exerted antiviral activity against HSV-2 with IC_50_ values in the range of 2 to 45 μM (26). Herein, we found that several *β*-carboline dimers possess better anti-PRV properties than monomers. These results further support that *β*-carboline dimers could serve as potential antiviral agents. Compounds **45** to **48** are bivalent 9-methyl-harmine with linkers containing four, five, six, or eight methylene units. With prolongation of the linker, anti-PRV activity decreases. However, **47** is an exception that does not inhibit PRV proliferation. The reasons for this remain unknown.

The stages of the virus life cycle usually consist of adsorption, entry, RNA/protein synthesis, assembly, and release. Quintana *et al.* found that 9N-methylharmine affects neither viral adsorption/entry events nor RNA synthesis during DENV proliferation. According to the quantification of intracellular and extracellular virus particles, they speculated that 9N-methylharmine could impair the maturation and release of DENV (10). Harmine, 9-methyl-norharmane, 9-methyl-harmane, and 6-methoxy-harmane mainly inhibit HSV proliferation at the post-entry stage (9, 27). Similarly, harmine also inhibits EV71 at the postentry stage (23). Herein, we found that **45** mainly interferes with the entry stage of the PRV life cycle, indicating a novel mechanism underlying the antiviral effects of *β*-carbolines. Although PRV and HSV both belong to the herpesviruses, the internalization mechanisms of PRV and HSV are different. Macropinocytosis is a major pathway of PRV entry into HeLa cells; the Na^+^/H^+^ exchanger (NHE), PI3K, p21-activated kinases (PaK1), and PKC contribute to the internalization of PRV (15). As for HSV, Devadas *et al.* (28) reported that HSV-1 internalization into epithelial cells requires NHE and PaK1, which could participate in macropinocytosis, but they also found that HSV-1 enters epithelial cells independently of PI3K and PKC. In contrast, Hilterbrand *et al.* found that both EIPA and NCS23766 (an inhibitor of Rac1) did not inhibit HSV-1 entry into C10 and CHO-HUVEM cells, implicating macropinocytosis is not the route of HSV-1 entry. However, they constructed a pseudotyped vesicular stomatitis virus (VSV) lacking its native glycoprotein G with four essential entry glycoproteins of HSV-1, gB, gH, gL, and gD (VSVΔG-BHLD), to study the features of HSV-1 entry. VSVΔG-BHLD entry into both C10 and CHO-HVEM cells was reduced by EIPA (an NHE inhibitor) and NSC23766 (a Rac1 inhibitor) but not by cytochalasin D (an actin polymerization inhibitor). Based on these results, they raised the interesting view that NHE and Rac1 could facilitate VSVΔG-BHLD entry into cells independently of their role in macropinocytosis (29). Thus, we regard the discrepant antiviral mode of action as related to the different entry pathways of viruses.

The *DYRK1A* gene is located on human chromosome 21q22.2, covering the Down syndrome critical region. Dysregulation of DYRK1A occurs in neurodegenerative diseases, Down syndrome, cancers, and diabetes (30, 31). Several reports showed that DYRK1A also participates in the proliferation of viruses. Inhibition of DYRK1A causes increased nuclear factor of activated T-cells binding to the HIV-1 long terminal repeat, thereby promoting viral transcription (32). DYRK1A can phosphorylate cyclin L2 at serine residues and control cyclin L2 expression, resulting in the restriction of HIV replication in macrophages (33). Huttereret *et al.* (14) provided the first evidence for the antiviral potential of DYRK inhibitors against herpesvirus. In this study, a DYRK1A inhibitor was found to suppress PRV proliferation. Through a series of assays, **45** was identified as a novel DYRK1A inhibitor that can inhibit macropinocytosis. DYRK1A knockdown has been proven to regulate macropinocytosis. However, the mechanisms underlying the regulatory effects of DYRK1A on macropinocytosis remain unknown. Previous work proved that DYRK1A inactivates glycogen synthase kinase 3β by phosphorylation at Thr356 (34). The canonical Wnt signaling pathway is emerging as a major regulator of endocytosis. Glycogen synthase kinase 3β inhibition was identified as a strong driver of macropinocytosis (35). Based on these findings, we speculate that DYRK1A may affect macropinocytosis by regulating glycogen synthase kinase 3β activity.

In summary, we identified 20 *β*-carboline derivatives that exhibited strong anti-PRV activity, with IC_50_ values in the range of 0.032 to 4.08 μM. Among them, **45** was the most effective compound and protected mice challenged with a lethal dose of PRV. Our results showed that **45** could inhibit the macropinocytosis-dependent entry of PRV by targeting DYRK1A. Overall, our findings may support the development of antiviral agents against PRV based on the *β*-carboline scaffold. These data provide mechanistic insights into the pharmacology of *β*-carboline derivatives. Finally, the present study reveals a potential drug target for the development of anti-PRV agents.

## Experimental procedures

### Ethics statement

All animal experiments were approved by the Animal Care and Use Committee of Northwest A&F University, China (approved no.: DY2022040) and were conducted following guidelines established by the Chinese Committee for Animal Experiments. All animals were euthanized by using carbon dioxide. All efforts were made to minimize suffering.

### *β*-carboline derivatives, reagents, cell lines, viruses, and antibodies

About 107 *β*-carboline derivatives were synthesized as previously described (36, 37, 38, 39, 40). The structures of all *β*-carboline derivatives are summarized in Tables S1–S3. EIPA, EHop-016, ML-141, AZ191, harmine, protease inhibitor cocktail, and phosphatase inhibitor cocktail I were purchased from TargetMol. Pronase, acyclovir, TNFα, and FITC–dextran 70 KDa (FITC-Dextran 70K) were purchased from Sigma–Aldrich. HeLa, Vero, BHK-21, and PK-15 cells (American Type Culture Collection) were cultured at 37 C in Dulbecco’s modified Eagle’s medium supplemented with 10% fetal bovine serum (Gibco). The PRV variant strain (SX-2015) and Bartha-K61 strain were propagated in Vero cells in our laboratory. Antibody against US9 was purchased from Developmental Studies Hybridoma Bank (Antibody Registry ID: AB_1553789). Antibodies against β-actin (catalog no.: 3700), DYRK1A (catalog no.: 2771), goat anti-rabbit immunoglobulin G (IgG) (catalog no.: 7074), goat antimouse IgG (catalog no.: 91196), and antimouse IgG (Alexa Fluor 488 conjugate; catalog no.: 4408) were purchased from Cell Signaling Technology. TRITC Phalloidin (catalog no.: 40734ES75) was purchased from Yeasen Biotechnology.

### Cytotoxicity assay

The cytotoxicity assay was performed as previously described (41).

### Plaque assay

The plaque assay was performed as previously described with some modifications (41). Briefly, HeLa cells were seeded in 24-well plates for 24 h. Then cells were infected with samples with a dilution of 10^−1^–10^−4^ at 37 °C for 1 h, washed with PBS, and covered with a medium containing methyl cellulose (1%). At 72 h postinfection, cells were fixed and stained with crystal violet solution for 20 min. Plaques were counted, and the virus titer was calculated.

### Time of addition assay

The time of addition assay was performed as previously reported to determine the mode of its activity with some modifications (24). Briefly, HeLa cells were infected with PRV (0.1 MOI). **45** (5 μM) was added at different time points: 0, 2, 4, 6, 8, and 12 h postinfection. At 24 h postinfection, the supernatant was collected, and viral yield was assessed by plaque assay. The cell lysates were harvested to measure protein expression by Western blot.

### Virucidal assay

PRV (1.5 × 10^6^ PFU/ml) was treated with **45** (50 μM) at room temperature for 2 h and then diluted to 150 PFU/100 μl. HeLa cells were infected with the pretreated virus mentioned previously (100 μl). After 1 h of adsorption, unbound virions were washed with PBS. And cells were then covered with medium-containing methylcellulose (1%). Plaques were visualized and counted by staining them with crystal violet after 72 h.

### Adsorption assay

The adsorption assay was conducted as previously described with some modifications (24). Briefly, HeLa cells were treated with **45** (5 μM) at 37 °C for 1 h, prechilled at 4 °C for 1 h, then infected with PRV (150 PFU) at 4 °C for 1 h. Finally, cells were washed three times with prechilled PBS and covered with medium-containing methylcellulose (1%). Finally, plaques were counted by staining them with crystal violet.

Meanwhile, another method was used to measure the binding viral particles. HeLa cells were treated with **45** (5 μM) at 37 °C for 1 h, prechilled at 4 °C for 1 h, then infected with PRV (50 MOI) at 4 °C for 1 h. Finally, cells were washed three times with prechilled PBS, fixed in 4% paraformaldehyde, and subjected to indirect immunofluorescence analysis.

### Entry assay

The entry assay was conducted as previously described with some modifications (24). Briefly, HeLa cells were treated with **45** (5 μM) at 37 °C for 1 h, prechilled at 4 °C for 1 h, then infected with PRV (50 MOI) at 4 °C for 1 h to allow viral adsorption. After that, (a) cells were washed with prechilled PBS and transferred into the incubator to allow viral entry. After 1 h, cells were washed with PBS and subjected to indirect immunofluorescence analysis; (b) viruses that had not entered were inactivated with a low pH buffer (40 mM Na citrate, 10 mM KCl, 135 mM NaCl, pH 3.0) and transferred into the incubator to allow viral entry. After 4 h, cells were washed with PBS and subjected to Western blot analysis.

### Transfection of siRNA

Control and DYRK1A siRNAs were obtained from RiboBio (China), and sequences are presented in Table S4. Transfections were performed according to the protocol of the Turbofect transfection reagent (Thermo Fisher).

### Molecular docking

Molecular docking was conducted using the Autodock 4, Autodock Vina, and AutodockTools-1.5.6 (42, 43). The crystal structure of DYRK1A (Protein Data Bank ID: 3ANR) was obtained from the Protein Data Bank (http://www.pdb.org). The 2D structure of **45** was generated using ChemBioDraw (CambridgeSoft Corporation), converted to 3D structure in the Sybyl-X 2.0 package, and the energy was optimized with the Tripos force field and the Gasteiger–Hückel method. The number of the final docking poses was set to 10. The structure with the highest affinity was selected to be a final structural model. The interactions were analyzed using the Discovery Studio Visualizer (BIOVIA).

### Drug affinity responsive target stability

DARTS was performed as previously described with some modifications (24). Briefly, HeLa cells were lysed with Mammalian Protein Extraction Reagent (M-PER; Thermo Scientific) supplemented with protease inhibitor cocktail (TargetMol) and phosphatase inhibitors (TargetMol). After centrifugation at 12,000 rpm for 20 min, protein concentration was quantified and diluted to 5 mg/ml, and 10× TNC buffer was added. Then cell lysates were incubated with DMSO or **45** (100 μM) at 4 °C overnight. All samples were divided into 50 μl aliquots in tubes and digested with indicated concentrations of pronase (Sigma–Aldrich) at room temperature for 15 min. The digestion was stopped by adding 20× protease inhibitor and SDS-PAGE sample loading buffer. Then the samples were boiled at 70 °C for 10 min and subjected to Western blot analysis.

### Cellular thermal shift assay

CETSA was performed as previously described with some modifications (24). Briefly, HeLa cells were lysed with M-PER (Thermo Scientific) supplemented with a protease inhibitor cocktail and phosphatase inhibitors. The lysates were then incubated with DMSO or **45** (100 μM) for 1 h at room temperature. The samples were divided into 50 μl aliquots in tubes and heated for 5 min at the indicated temperature, followed by incubation on ice for 10 min. The samples were then added with SDS-PAGE sample loading buffer, boiled at 70 °C for 10 min, and subjected to Western blot analysis.

### DNA/RNA extraction and quantitative real-time qPCR

Viral DNA extraction was performed using a TIANamp Virus DNA/RNA kit according to the manufacturer's instructions. The RNA extraction and real-time qPCR were performed as previously described (24). The primers used in this study are listed in Table S4.

### FITC–dextran uptake assays

HeLa cells were untreated or treated with the **45** (5 μM) for 1.5 h, incubated with 5 mg/ml FITC–dextran 70K for 1 h, washed three times with cold PBS, and washed twice with low pH buffer (0.1 M sodium acetate, 0.05 M NaCl, pH 5.5). Then the samples were fixed in 4% paraformaldehyde and subjected to indirect immunofluorescence analysis.

### Western blot analysis

Western blot analysis was performed by standard procedures as previously described (24). The intensities of target bands were analyzed using ImageJ software (NIH).

### Safety assessment

A total of 30 female-specific pathogen-free KM mice (6–8 weeks) were purchased from the Chengdu Dossy Experimental Animals Co and randomly divided into three groups. Mice were treated with DMSO or **45** (1 mg/kg or 10 mg/kg). The health and behavior of all mice were monitored daily every 12 h throughout the experimental period. All mice were weighed and euthanized at 14 days postinoculation. The livers, spleens, and kidneys were collected and weighed.

### *In vivo* antiviral activities of compound

A total of 100 female-specific pathogen-free KM mice (6–8 weeks) were purchased from the Chengdu Dossy Experimental Animals Co Ltd and randomly divided into five groups. The treatments for each group are listed in Table S5. In group I, mice were intraperitoneally injected with Dulbecco’s modified Eagle’s medium along with DMSO. In group II–V, all mice were intraperitoneally injected with PRV (1 × 10^3^ PFU or 1 × 10^4^ PFU) along with DMSO (group II and IV) or compound **45** (groups III and V). Five mice in each group were euthanized, and the spleen tissues were collected to detect viral loads by qPCR at 72 h postinoculation. Five mice in each group were euthanized, and the spleen tissues were collected and subjected to histopathological analysis at 72 h postinoculation. The health and behavior of the mice were monitored every 12 h throughout the experimental period. Clinical scores were calculated by the following criteria: posture normal = 0; mild neurological symptoms = 1: excitation, unrest, occasional itching; the absence of neurological symptoms = 2: ataxia, severe pruritus, and self-mutilation; death = 3.

### Indirect immunofluorescence

Indirect immunofluorescence was performed as previously described (24). Briefly, HeLa cells were treated with the indicated reagents and infected with PRV, washed three times, and fixed in 4% paraformaldehyde, permeabilized with 0.5% Triton X-100 for 20 min, blocked in PBS with Tween-20 containing 5% bovine serum albumin at 37 °C for 1 h, and then incubated with antibody against US9 at 37 °C for 1 h. Cell nuclei were stained with 4′,6-diamidino-2-phenylindole (1 μg/ml) for 10 min. Actin filaments were stained with TRITC–phalloidin (2 μg/ml) at 37 °C for 30 min. Cells were visualized using a confocal laser-scanning microscope (LEICA TCS SP8).

### Histopathological analysis

Five mice were randomly selected from each group at 3 days postinoculation. Spleens were collected and preserved in 4% paraformaldehyde, embedded by paraffin, and sectioned. The 5 μm sections were stained with hematoxylin and eosin. All samples were observed and photographed using an optical microscope (Ni-U; Nikon).

### Statistical analysis

All the experiments were applied in triplicate, and each experiment was independently repeated at least three times. Results were represented as means ± standard deviations of the mean. The values of CC_50_ or IC_50_ were calculated using GraphPad Prism 6.0 software (GraphPad Software, Inc). Statistical analysis was done by two-tailed Student’s *t* test using GraphPad Prism 6.0 software. Statistical significance: ns, not significant, ∗*p* < 0.05, ∗∗*p* < 0.01, and ∗∗∗*p* < 0.001.

## Data availability

All the data have been included in the article.

## Supporting information

This article contains supporting information.

## Conflict of interest

The authors declare that they have no conflicts of interest with the contents of this article.
