# Supplementary material for: A bivalent β-carboline derivative inhibits macropinocytosis-dependent entry of pseudorabies virus by targeting the kinase DYRK1A
Source: J Biol Chem. 2023 Mar 12;299(4):104605. doi: 10.1016/j.jbc.2023.104605 (PMC10140166; doi:10.1016/j.jbc.2023.104605)
Supplement: Supplemental Tables S1–S5 and Figures S1–S5 [file mmc1.docx]

Supporting information

**A bivalent *β*-carboline derivative inhibits macropinocytosis-dependent entry of pseudorabies virus by targeting the kinase DYRK1A**

Chongyang Wang^1,†^, Ruochen Hu^1,†^, Ting Wang^1^, Liuyuan Duan^1^, Qili Hou^1^, Junru Wang^2,*^, Zengqi Yang^1,*^

^1^ College of Veterinary Medicine, Northwest A&F University, Xianyang 712100, China;

^2^ College of Chemistry and Pharmacy, Northwest A&F University, Xianyang 712100, China

^†^These authors contributed equally to this work.

^*^Corresponding author: Junru Wang, wangjunru@nwsuaf.edu.cn; Zengqi Yang, yzq1106@nwsuaf.edu.cn.

**Table S1**, **S2**, and **S3** provide the structures of *β*-carboline derivatives used in this study.

**Table S4** provides the sequence of primers and siRNAs used in this study.

**Table S5** provides the animal grouping and treatments in this study.

**Figure S1**, **S2**, and **S3** provide the results of the primary screening of *β*-carboline monomers, *β*-carboline dimers, and canthin-6-one analogs.

**Figure S4** provides the results of antiviral activities of effective *β*-carboline derivatives detected by qPCR.

**Figure S5** provides the results of antiviral activities of effective *β*-carboline derivatives against Bartha-K61.

**Table S1**

The numbers and structures of *β*-carboline monomers

| **No.** | **Structure** | **No.** | **Structure** |
| --- | --- | --- | --- |
| **1** | **** | **2** | **** |
| **3** | **** | **4** | **** |
| **5** | **** | **6** | **** |
| **7** | **** | **8** | **** |
| **9** | **** | **10** | **** |
| **11** | **** | **12** | **** |
| **13** | **** | **14** | **** |
| **15** | **** | **16** | **** |
| **17** | **** | **18** | **** |
| **19** | **** | **20** | **** |
| **21** | **** | **22** | **** |
| **23** | **** | **24** | **** |
| **25** | **** |  |  |

**Table S2**

The numbers and structures of *β*-carboline dimers

| **No.** **: R** | Structure |
| --- | --- |
| **26**：Butane-1,4-diyl  **27**：Pentane-1,5-diyl  **28**：Hexan-1,6-diyl  **29**：Octan-1,8-diyl  **30**：*p*-Xylene-α,α’-diyl |  |
| **31**：Butane-1,4-diyl  **32**：Pentane-1,5-diyl  **33**：Hexan-1,6-diyl  **34**：Octan-1,8-diyl  **35**：*p*-Xylene-α,α’-diyl |  |
| **36**：Butane-1,4-diyl  **37**：Pentane-1,5-diyl  **38**：Hexan-1,6-diyl  **39**：Octan-1,8-diyl  **40**：*p*-Xylene-α,α’-diyl |  |
| **41**：Butane-1,4-diyl  **42**：Pentane-1,5-diyl  **43**：Hexan-1,6-diyl  **44**：Octan-1,8-diyl |  |
| **45**：Butane-1,4-diyl  **46**：Pentane-1,5-diyl  **47**：Hexan-1,6-diyl  **48**：Octan-1,8-diyl |  |
| **49**：Butane-1,4-diyl  **50**：Pentane-1,5-diyl  **51**：Hexan-1,6-diyl  **52**：Octan-1,8-diyl |  |
| **53**：Butane-1,4-diyl  **54**：Pentane-1,5-diyl  **55**：Hexan-1,6-diyl  **56**：Octan-1,8-diyl |  |
| **57**：Butane-1,4-diyl  **58**：Pentane-1,5-diyl  **59**：Hexan-1,6-diyl  **60**：Octan-1,8-diyl |  |
| **61**：Butane-1,4-diyl  **62**：Pentane-1,5-diyl  **63**：Hexan-1,6-diyl  **64**：Octan-1,8-diyl |  |

**Table S3**

The numbers and structures of canthin-6-one analogs

| **No.** | Structure | **No.** | Structure |
| --- | --- | --- | --- |
| **65** |  | **66** |  |
| **67** |  | **68** |  |
| **69** |  | **70** |  |
| **71** |  | **72** |  |
| **73** |  | **74** |  |
| **75** |  | **76** |  |
| **77** |  | **78** |  |
| **79** |  | **80** |  |
| **81** |  | **82** |  |
| **83** |  | **84** |  |
| **85** |  | **86** |  |
| **87** |  | **88** |  |
| **89** |  | **90** |  |
| **91** |  | **92** |  |
| **93** |  | **94** |  |
| **95** |  | **96** |  |
| **97** |  | **98** |  |
| **99** |  | **100** |  |
| **101** |  | **102** |  |
| **103** |  | **104** |  |
| **105** |  | **106** |  |
| **107** |  |  |  |

**Table S4**

The sequence of primers and siRNAs used in this study

| Gene name | Forward primer（5’-3’） | Reverse primer（5’-3’） |
| --- | --- | --- |
| Primers | | |
| DYRK1A | TGCCATTGATATGTGGTCCCT | TCAAGAATATGAGCAGGTGGA |
| PRV-gE | TCTGCGTGCTGTGiCTCCC | TCGTCGCCGTCGTAGTAG |
| β-actin | TGGACATCCGCAAAGACCTGT | GGAGTACTTGCGCTCAGGAGG |
| siRNA | | |
| siNC | UUCUCCGAACGUGUCACGUTT | |
| siDYRK1A01 | GGAGACGATTCTAGTCATA | |
| siDYRK1A02 | CCAACAGGTTTCTGCCTTA | |
| siDYRK1A03 | GCAAGAAAGTTCTTTGAGA | |

**Table S5**

Animal grouping and treatments

| Group | Mice number | Virus titer | Compd.**45** |
| --- | --- | --- | --- |
| I | 20 | - | - |
| II | 20 | 1×10^3^PFU | - |
| III | 20 | 1×10^3^PFU | 0.2 mg/kg |
| IV | 20 | 1×10^4^PFU | - |
| V | 20 | 1×10^4^PFU | 0.2 mg/kg |


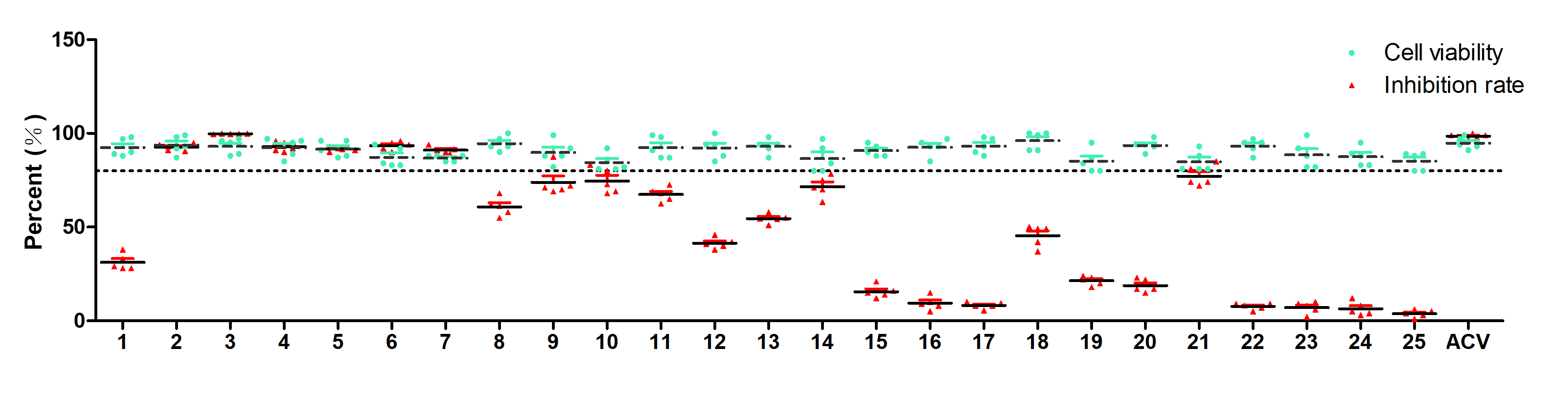


**Figure S1. Primary screening of *β*-carboline monomers with antiviral activities against PRV.**

Cell viability was assessed by CCK-8 in HeLa cells treated with *β*-carboline monomers at 48 h post-incubation. HeLa cells were infected with PRV (0.1 MOI) and covered with DMEM containing *β*-carboline monomers (5 μM). At 24 h post-infection, the virus yield in the supernatant was measured by plaque assay and the inhibition rate was calculated.


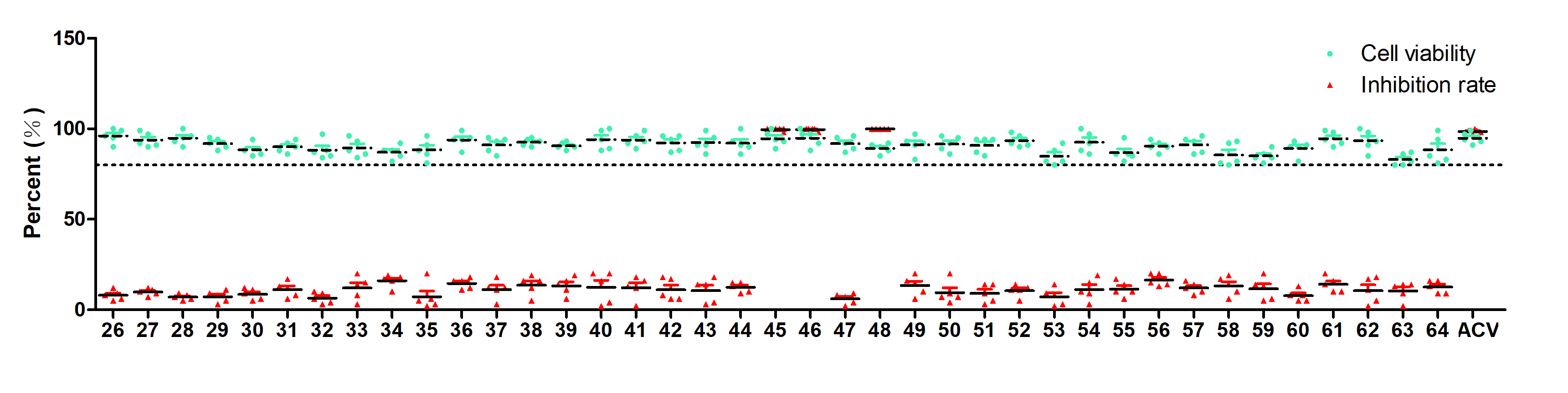


**Figure S2. Primary screening of *β*-carboline dimers with antiviral activities against PRV.**

Cell viability was assessed by CCK-8 in HeLa cells treated with *β*-carboline dimers at 48 h post-incubation. HeLa cells were infected with PRV (0.1 MOI) and covered with DMEM containing *β*-carboline dimers (5 μM). At 24 h post-infection, the virus yield in the supernatant was measured by plaque assay and the inhibition rate was calculated.


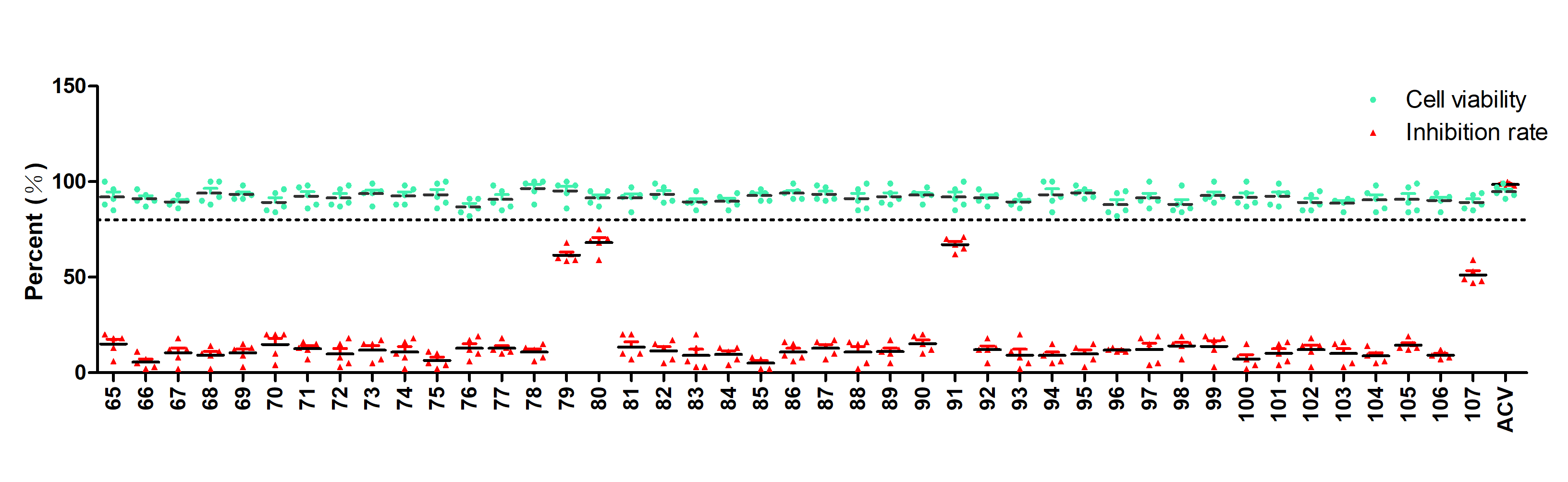


**Figure S3. Primary screening of canthin-6-one analogs with antiviral activities against PRV.**

Cell viability was assessed by CCK-8 in HeLa cells treated with canthin-6-one analogs at 48 h post-incubation. HeLa cells were infected with PRV (0.1 MOI) and covered with DMEM containing canthin-6-one analogs (5 μM). At 24 h post-infection, the virus yield in the supernatant was measured by plaque assay and the inhibition rate was calculated.


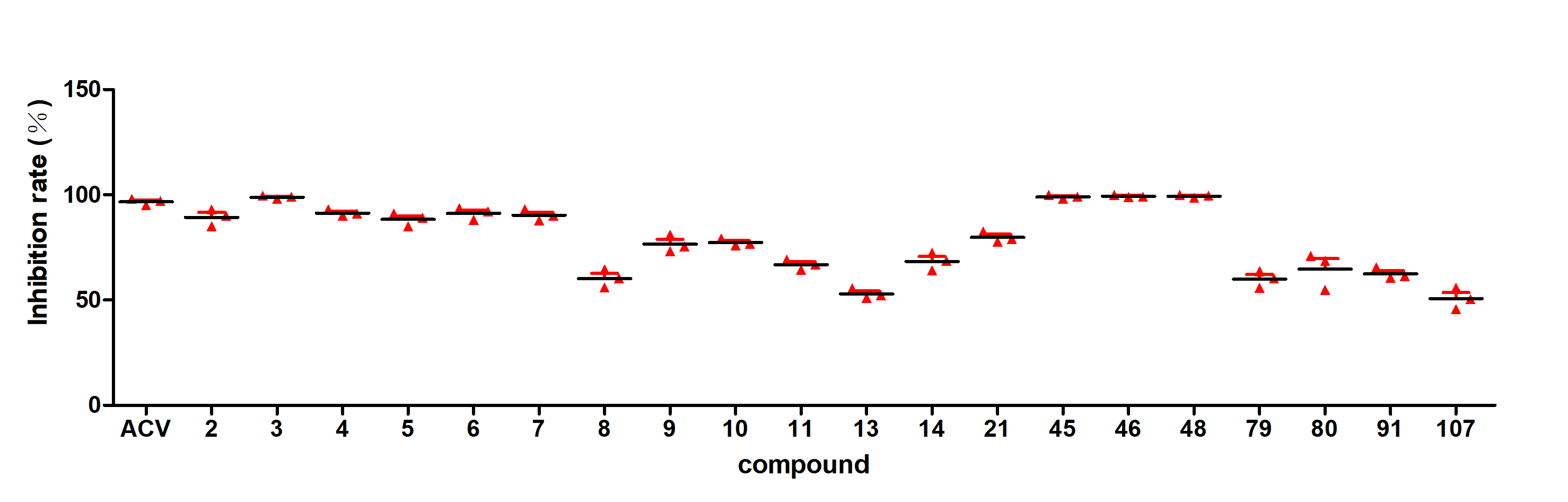
**Figure S4.** **The antiviral activities of effective compounds.**

HeLa cells were infected with PRV (0.1 MOI) and covered with DMEM containing *β*-carboline derivatives (5 μM). At 24 h post-infection, cells were harvested to detect viral DNA by qPCR and the inhibition rate was calculated.


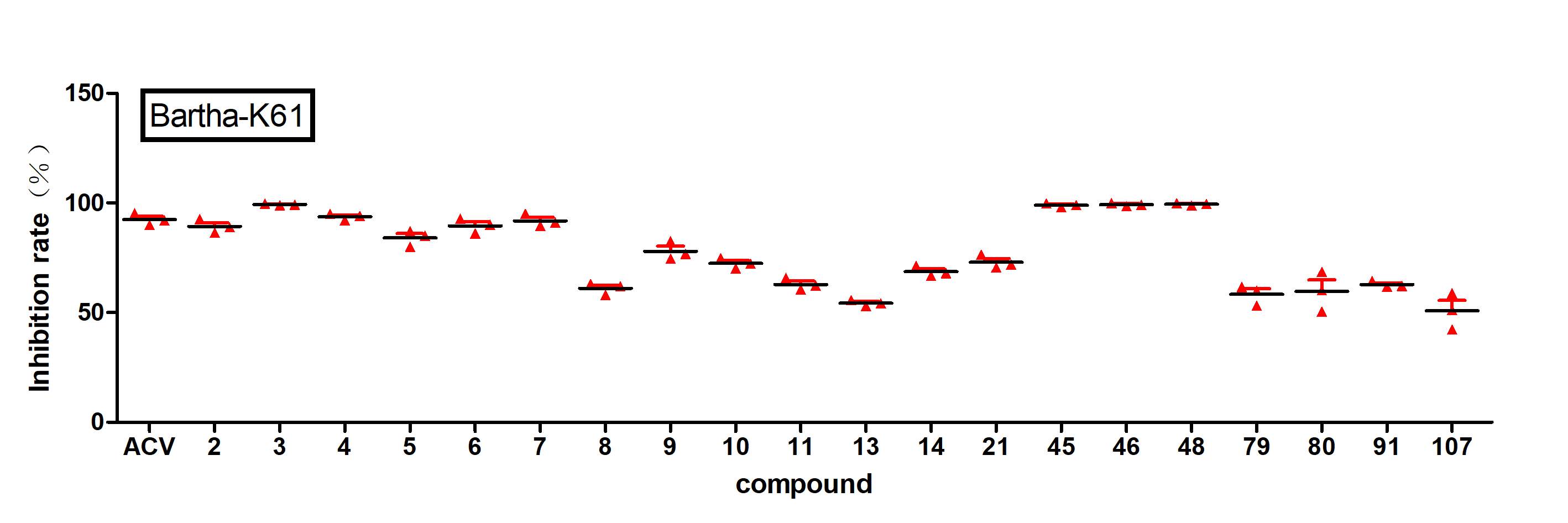


**Figure S5. The antiviral activities of effective compounds against Bartha-K61.**

HeLa cells were infected with Bartha-K61 (0.1 MOI) and covered with DMEM containing *β*-carboline derivatives (5 μM). At 24 h post-infection, the virus yield in the supernatant was measured by plaque assay and the inhibition rate was calculated.
